# Variable VlsE Is Critical for Host Reinfection by the Lyme Disease Spirochete

**DOI:** 10.1371/journal.pone.0061226

**Published:** 2013-04-08

**Authors:** Artem S. Rogovskyy, Troy Bankhead

**Affiliations:** 1 Department of Veterinary Microbiology and Pathology, Washington State University, Pullman, Washington, United States of America; 2 Paul G. Allen School for Global Animal Health, Washington State University, Pullman, Washington, United States of America; University of Kentucky College of Medicine, United States of America

## Abstract

Many pathogens make use of antigenic variation as a way to evade the host immune response. A key mechanism for immune evasion and persistent infection by the Lyme disease spirochete, *Borrelia burgdorferi*, is antigenic variation of the VlsE surface protein. Recombination results in changes in the VlsE surface protein that prevent recognition by VlsE-specific antibodies in the infected host. Despite the presence of a substantial number of additional proteins residing on the bacterial surface, VlsE is the only known antigen that exhibits ongoing variation of its surface epitopes. This suggests that *B. burgdorferi* may utilize a VlsE-mediated system for immune avoidance of its surface antigens. To address this, the requirement of VlsE for host reinfection by the Lyme disease pathogen was investigated. Host-adapted wild type and VlsE mutant spirochetes were used to reinfect immunocompetent mice that had naturally cleared an infection with a VlsE-deficient clone. Our results demonstrate that variable VlsE is necessary for reinfection by *B. burgdorferi*, and this ability is directly related to evasion of the host antibody response. Moreover, the data presented here raise the possibility that VlsE prevents recognition of *B. burgdorferi* surface antigens from host antibodies. Overall, our findings represent a significant advance in our knowledge of immune evasion by *B. burgdorferi*, and provide insight to the possible mechanisms involved in VlsE-mediated immune avoidance.

## Introduction

*Borrelia burgdorferi* is the causative agent of the multisystem disease known as Lyme borreliosis, which is currently the most prevalent vector-borne disease in North America [1], [2], [3]. Infection with this spirochete can be severely debilitating to both animals and humans, resulting in long-term manifestations including arthritis, carditis, and neurological problems [4]. Although persistent infection can last from months to years due to avoidance of the host immune response by the pathogen, early infection can usually be cleared with antibiotic treatment. Surprisingly, reinfection occurs fairly regularly in post-treatment patients that have successfully cleared initial infection, suggesting that individuals treated for early Lyme disease continue to remain at risk for reinfection [5], [6], [7], [8], [9], [10], [11], [12], [13]. The incidence of reinfection has been shown to be as high as 15% over a five-year study period (a rate of 3% per year), and clinical manifestations seem to be identical to those of initial infection [6].

Key to the successful immune evasion tactics of *B. burgdorferi* is recombination at the *vls* locus located at the right telomeric end of a 28-kilobase linear plasmid (lp28-1) in the B31 strain [14], [15], [16]. Recombinational switching at the *vls* locus results in sequence variation of the surface lipoprotein, VlsE, which alters its antigenic properties and allows the spirochete to evade the host's antibody-mediated response [16], [17], [18]. Evidence for the role of the *vls* system in immune avoidance was first provided by studies involving the *vls*-resident plasmid, lp28-1 [19], [20]. Clones lacking lp28-1 were shown to exhibit an intermediate infectivity phenotype whereby these spirochetes were able to disseminate to tissue sites but were unable to persist in the murine host. These same clones are capable of long-term survival in severe-combined immunodeficient (SCID) mice that lack an effective antibody response [21], [22]. lp28-1-deficient isolates also grow normally in a dialysis membrane chamber implanted in the peritoneal cavity of rats, where exposure to either antibodies or immune cells is restricted [22]. Moreover, immunocompetent mice infected with an lp28-1-deficient clone complemented with only the *vlsE* gene (*sans* the *vls* silent cassettes) are able to clear infection, demonstrating that it is not the mere presence of VlsE that provides the capacity for persistent infection, but rather the ability to undergo *vls* recombination to produce VlsE variants [23]. Finally, spirochetes that lack only the *vls* locus due to telomere-mediated removal are completely cleared from immunocompetent C3H mice by 21 days post infection [24], confirming the hypothesis that *vls* recombination functions to evade the humoral immune response in the mouse host [14], [16], [25], [26].

Recombination events within *vlsE* have been detected as early as four days post infection in mice, and continue to occur throughout infection [18], [27], [28]. Moreover, antibodies specific for the variable regions of VlsE were shown to be produced during experimental infection of mice [29]. Interestingly, VlsE antigenic switching in *B. burgdorferi* is only detectable during mammalian infections, suggesting that host factors may be required to enhance the antigenic variation process [16], [17], [18], [26], [30], [31]. A role for the VlsE protein other than antigenic variation is not currently known, but it has been proposed that the protein might function in other forms of immune evasion [32], [33].

Although a number of other surface proteins exist that are immunogenic, VlsE is the only known *B. burgdorferi* antigen that exhibits active variation of its surface epitopes. This fact may suggest that *B. burgdorferi* uses a specialized VlsE-mediated system for immune avoidance of its surface antigens. Many pathogens utilize their antigenically variable proteins in a number of ways as an evasion strategy, and models have been suggested for how VlsE might become the primary target for the host immune response [24], [33]. One possibility is that VlsE may act as a shield to obscure the epitopes of other surface antigens. A precedent for this type of interaction has been demonstrated in studies with the *B. burgdorferi* protein P66, in which the protein is protected from antibodies and proteolytic cleavage in spirochetes expressing high levels of the outer surface protein, OspA [34]. It has also been proposed that VlsE might be a T-cell independent antigen that could directly stimulate B cells [24], [33]. The resulting humoral response generated by VlsE may serve to override antibody production against other potential surface antigens in such a way that antibodies to non-VlsE surface antigens are produced at insufficient titers in order to clear the *Borrelia* infection.

To date, a link between the capacity of *B. burgdorferi* for host reinfection and the antigenically variable VlsE protein has not been examined in detail. In the current study, we utilized host-adapted wild type and VlsE-deficient clones to infect mice with an active humoral response to *B. burgdorferi* in order to address the question of VlsE-mediated immune evasion. We report for the first time that variable VlsE is critical for establishing murine reinfection by *B. burgdorferi*. Moreover, our findings suggest that host reinfection occurs through escape of non-VlsE surface antigens from humoral immune surveillance, possibly through a VlsE-dependent mechanism.

## Results

### Generation and characterization of a *B. burgdorferi* mutant expressing non-variable VlsE

A recent study attempted to address the role of VlsE in host reinfection through the use of sera from infected mice [24], however the results from this study were ultimately inconclusive. The inherent problem with these experiments is that *in vitro*-cultured *B. burgdorferi* were utilized, which have been shown to have a 32-fold reduction in VlsE expression levels relative to those measured during murine infection [35]. Unlike these highly susceptible *in vitro*-grown spirochetes, *B. burgdorferi* that have adapted within either the feeding tick or animal host have been demonstrated to be relatively invulnerable to the protective effects of immune sera [36], [37]. For this reason, we chose to use host-adapted spirochetes in order to re-examine whether reinfection of a mouse host by *B. burgdorferi* requires the presence of the VlsE protein. To obtain host-adapted spirochetes for this study, ear tissues from severe combined immunodeficient (SCID) mice infected with either wild-type or *vlsE* mutant *B. burgdorferi* clones were harvested. These ear tissues containing host-adapted *B. burgdorferi* were then transplanted via subcutaneous incisions in order to infect or reinfect mice.

**Figure 1:**
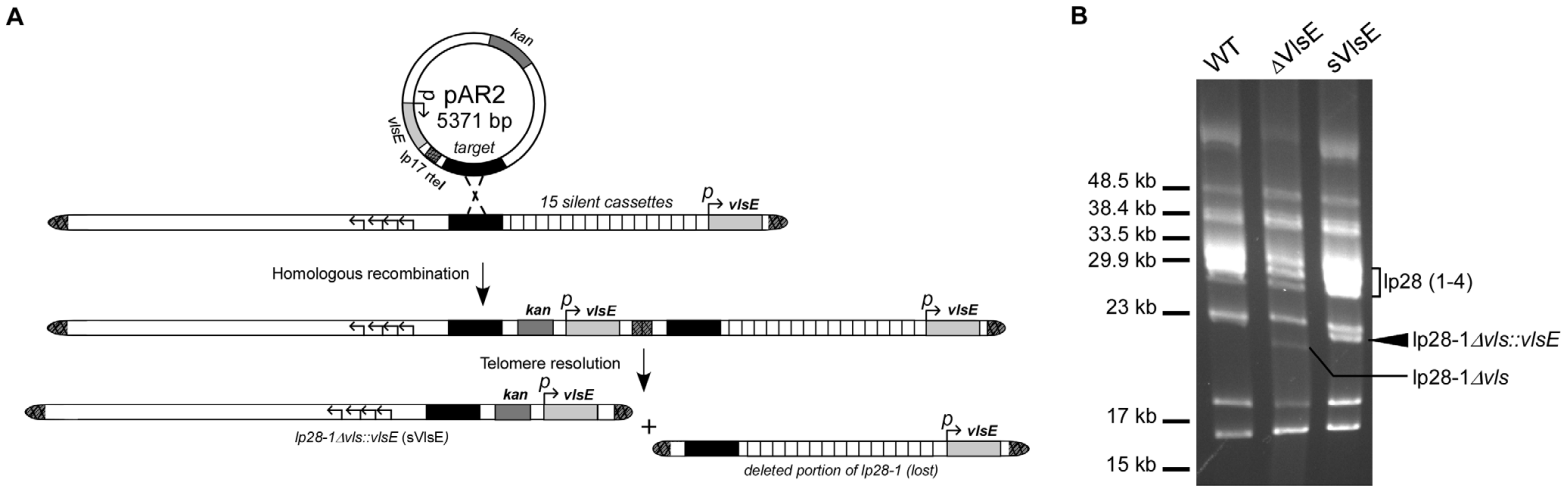
Generation of *B. burgdorferi* mutant with non-switchable *vlsE in cis*. A) Schematic illustrating the replacement of the *vls* locus with a non-switchable *vlsE* gene. The targeted deletion construct, pAR2, is inserted into lp28-1 via homologous recombination at the target DNA sequence. Following telomere resolution by endogenous ResT, the right end of lp28-1 containing the 15 silent cassettes and *vlsE* expression site is deleted. The resultant truncated plasmid, lp28-1Δ*vls*::*vlsE*, contained *vlsE* with native basal promoter (denoted as *p*) and kanamycin-resistant gene (*kan*). The four leftward facing arrows represent the genes required for autonomous replication of lp28-1. Telomere regions and replicated telomere (*rtel*) are indicated as hatched regions. B) Analysis of the lp28-1Δ*vls*::*vlsE* construct by field inversion gel electrophoresis. Genomic DNA from WT, ΔVlsE, and sVlsE are shown in lanes 1, 2, and 3, respectively. Positions of lp28-1Δ*vls*::*vlsE* and lp28-1Δ*vls* are shown by the arrowhead and angled line, respectively. The positions of DNA markers are indicated on the left.

In order to determine whether variable or static VlsE could provide a capacity for reinfection, we first set out to generate a *B. burgdorferi* mutant clone containing a non-switchable *vlsE* gene on lp28-1. To generate this “static” VlsE mutant clone (sVlsE), we utilized a targeted deletion strategy that resulted in the replacement of the entire *vls* locus with a copy of only the *vlsE* gene and native basal promoter. As shown in Figure 1A, the targeted deletion plasmid contains a kanamycin-resistant cassette (*kan*), a replicated telomere (*rtel*), and a target DNA sequence identical to a 1.2 kb stretch of lp28-1 just upstream of the *vls* locus in the B31-A3 wild type clone [38]. Homologous recombination at the DNA target sequence followed by telomere resolution by endogenous ResT leads to deletion of the 15 silent cassettes and native *vlsE* expression site, which is replaced by the *kan* gene and a newly introduced copy of *vlsE* with its native promoter. This *vlsE* gene is unable to undergo *vls* recombination due to the absence of the 15 silent cassettes, and therefore *B. burgdorferi* clones carrying this non-switchable *vlsE* will only express a single static version of the VlsE lipoprotein.

**Figure 2:**
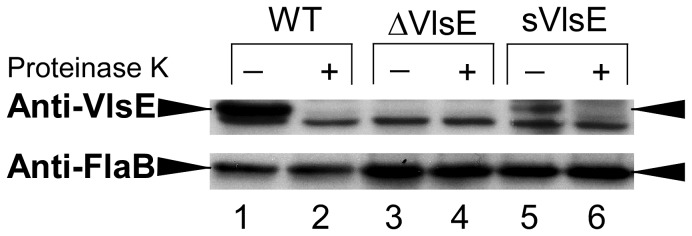
Expression and surface localization of VlsE by *B. burgdorferi* clones. Intact spirochetes were treated with or without proteinase K for 40 min followed by SDS-polyacrylamide gel electrophoresis (10^8^ cells/lane) and Western blotting. Two identical blots were processed with anti-VlsE or anti-FlaB antibodies. Lanes 1, 3, and 5 show that VlsE is only expressed by the WT and sVlsE clones, but not by the ΔVlsE clone. Treatment with proteinase K dramatically reduced VlsE immunostaining for the WT and sVlsE clones, but had no effect on levels of the periplasmic protein, FlaB. A non-specific band that remains after proteinase K digestion can also be seen in the anti-VlsE blot.

Overall, ten kanamycin-resistant transformants were recovered and PCR screened for the presence of the *kan* gene. Several clones were selected and further analyzed by PCR to ensure retention of *B. burgdorferi* plasmids, including those essential for infectivity [39]. One sVlsE clone that contained the full plasmid profile, with the exception of cp9, was chosen for further analyses (Table 1). The cp9 plasmid is normally absent from the parental B31-A3 wild type clone, and is not necessary for infection or pathogenesis [38]. The truncation of lp28-1 was confirmed by field inversion gel analysis (Figure 1B) and verified by Southern blot (data not shown). Expression and surface localization of VlsE by the sVlsE clone was confirmed by surface proteolysis via Western blot analysis (Figure 2), which showed lower overall expression than that of the wild type (WT). As shown in Table 2, the sVlsE clone was found to be infectious in both immunocompetent C3H/HeNHsd (C3H) and immunodeficient SCID mice. Similar to published studies involving previously characterized non-switchable VlsE mutants [16], [24], the sVlsE clone was not able to establish persistent infection in immunocompetent C3H mice, but could maintain long-term infection in SCID mice. Similarly and consistent with a previous study, a clone lacking the entire *vls* locus (ΔVlsE) exhibited persistent infection only in immunodeficient SCID animals [24].

**Table 1:** *Borrelia burgdorferi* clones used in the study.

*B. burgdorferi* B31 clone	Missing plasmid(s)	*vls2-16* a	*vlsE*	Reference
A3 (WT)	cp9	+	+	[38]
A3 lp28-1Δ*vls* (ΔVlsE)	cp9	−	−	[24]
A3 lp28-1Δ*vls*::*vlsE* (sVlsE)	cp9	−	+	This study

a *vls2-16* denotes silent cassettes of the *vls* locus.

**Table 2:** Infectivity of *in vitro*-grown VlsE mutants in naïve SCID and C3H mice.

Tissue collected (at day post infection)	Naïve SCID mice infected with			Naïve C3H mice infected with		
	WT	ΔVlsE	sVlsE	WT	ΔVlsE	sVlsE
Blood (day 7)	3/3^a^	3/3	3/3	4/4	5/5	5/5
Ear (day 14)	3/3	3/3	3/3	3/4	3/5	4/5
Ear (day 21)	3/3	3/3	3/3	4/4	0/5	0/5
Ear (day 28)	3/3	3/3	3/3	4/4	0/5	0/5

a Values listed correspond to numbers of cultures positive/numbers tested.

Finally, in order to confirm the infectivity of host-adapted *B. burgdorferi* clones to be used for this study, SCID ear tissues containing either the ΔVlsE mutant clone, sVlsE mutant clone, or WT control were transplanted into naïve C3H mice. As shown in Table 3, blood collected and cultured for spirochetes at day 7 post infection demonstrated that all mice had been successfully infected with each of the host-adapted *B. burgdorferi* clones. Ear tissues collected at days 14, 21 and 28 post infection showed that mice infected with WT were persistently infected, while all mice infected with either of the VlsE-mutant clones were cleared of infection by day 21 post infection. With an infectious clone expressing a static form of VlsE now generated and the infectivity of host-adapted clones verified, the requirement of VlsE for host reinfection was assessed.

**Table 3:** Infectivity of host-adapted VlsE mutants in naïve C3H mice.

Tissue collected (at day post infection)	Naïve C3H mice infected via tissue transplantation with^a^		
	ha WT^b^	ha ΔVlsE	ha sVlsE
Blood (day 7)	3/3	3/3	3/3
Ear (day 14)	3/3	1/3	2/3
Ear (day 21)	3/3	0/3	0/3
Ear (day 28)	3/3	0/3	0/3

a Values listed correspond to numbers of cultures positive/numbers tested.

b ha denotes host-adapted clone.

### Variable VlsE is required for host reinfection by *B. burgdorferi*

**Figure 3:**
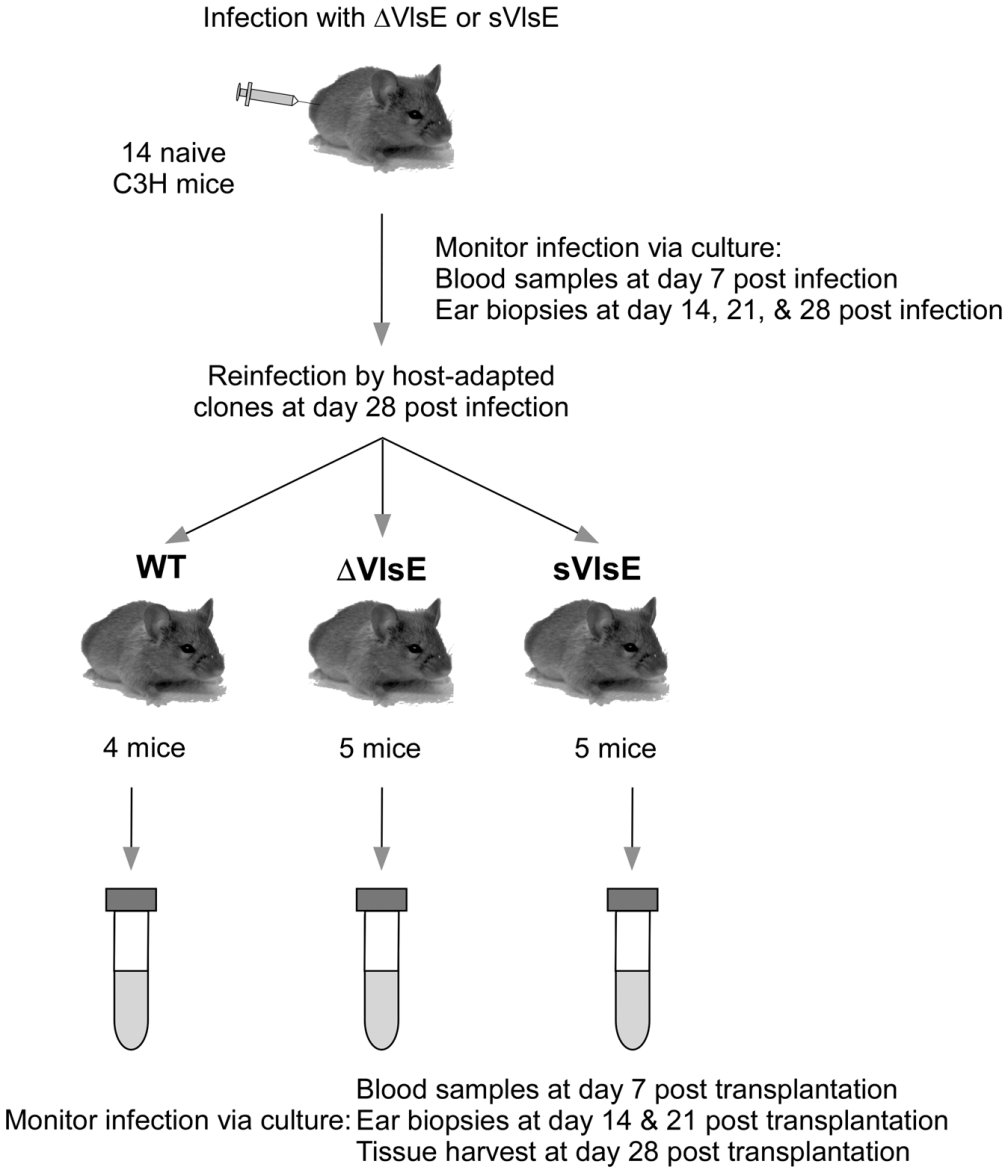
Experimental design to assay for a VlsE requirement for host reinfection. C3H mice were initially infected with *in vitro*-grown ΔVlsE or sVlsE clones. At day 28 post infection, a time at which spirochetes had been cleared due to a host antibody response, animals were divided into three groups of 4 or 5 mice each and reinfected with either host-adapted WT, ΔVlsE, or sVlsE via tissue transplantation. Blood samples, ear biopsies and other harvested tissues (heart, bladder, and joint) were collected at indicated time points post transplantation and cultured to monitor the reinfection outcome.

In order to determine the requirement of VlsE for host reinfection by *B. burgdorferi*, an experimental scheme was used in which 14 immunologically-naïve C3H mice were initially infected with either the ΔVlsE or sVlsE mutant clone (Figure 3). Infection of mice occurred via needle inoculation, and the progress of infection was monitored weekly. As expected, all animals were successfully infected as shown by positive cultures of blood taken at day 7 post infection, and all mice cleared infection by day 21 post infection as detected by negative ear cultures (data not shown; [24]). At day 28 post infection, the VlsE-naïve mice were separated into three groups, and then challenged with either host-adapted WT, ΔVlsE or sVlsE via tissue transplantation to assay for the ability to reinfect mice (see Figure 3).

**Figure 4:**
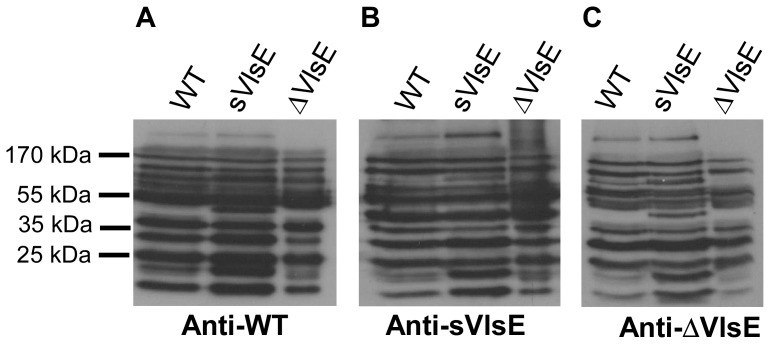
Analysis of *B. burgdorferi*-specific immune sera by immunoblotting. The whole-cell lysates of *B. burgdorferi* WT, sVlsE, and ΔVlsE clones (10^6^ cells/lane) were treated with anti-WT, anti-sVlsE, or anti-ΔVlsE (panel A, B, and C, respectively) immune sera collected from C3H mice at day 28 post infection. Preimmune sera-treated immunoblot had no immune banding (not shown).

The results from this experiment showed that host-adapted WT was able to reinfect all mice that had cleared ΔVlsE-induced infection (4 out of 4) as determined by positive cultures of blood samples drawn at day 7 post transplantation (group ΔVlsE-WT; Table 4). Moreover, ear tissues obtained from all animals at day 21 and 28 post transplantation were culture positive for the WT clone, indicating that the mice became persistently infected. WT spirochetes were also cultured from heart, bladder and joint tissues harvested at day 28 post transplantation, demonstrating tissue dissemination. In contrast, the ΔVlsE clone was not able to reinfect any mice originally infected with and cleared of this same clone (group ΔVlsE-ΔVlsE; Table 4). Immunoblots of WT, sVlsE, or ΔVlsE whole-cell lysates that were blotted with *B. burgdorferi* clone-specific immune sera collected at day 28 post infection displayed similar antibody response patterns (Figure 4). Thus, the difference in the ability of *B. burgdorferi* clones to reinfect does not seem to be attributed to any noticeable difference in the total *Borrelia*-specific antibody response. Together, these results suggest that the immune response of these mice to non-VlsE surface antigens can sufficiently block reinfection by a *B. burgdorferi* clone lacking VlsE, but is unable to prevent reinfection by the WT clone containing VlsE. However, the sVlsE clone was not recovered from any mouse (group ΔVlsE-sVlsE; Table 4) that had previously cleared infection with the ΔVlsE clone, suggesting that the presence of a non-switchable form of VlsE in these spirochetes does not provide the capacity for reinfection. Thus, this finding may indicate that it is not the mere presence of VlsE that is necessary to allow host reinfection, but rather a variable form of the protein.

**Table 4:** Assessment of reinfection in immunologically VlsE-naïve and VlsE-exposed C3H mice.

Tissue collected (at day post challenge)	ΔVlsE-cleared C3H mice reinfected with^a^			sVlsE-cleared C3H mice reinfected with		
	ha WT^b^ (ΔVlsE-WT)	ha ΔVlsE (ΔVlsE-ΔVlsE)	ha sVlsE (ΔVlsE -sVlsE)	ha WT (sVlsE-WT)	ha ΔVlsE (sVlsE-ΔVlsE)	ha sVlsE (sVlsE -sVlsE)
Blood (day 7)	4/4	0/5	0/5	4/4	0/5	0/5
Ear (day 14)	0/4	0/5	0/5	4/4	0/5	0/5
Ear (day 21)	4/4	0/5	0/5	4/4	0/5	0/5
Ear and other tissues^c^ (day 28)	4/4	0/5	0/5	4/4	0/5	0/5

a Values listed correspond to numbers of cultures positive/numbers tested.

b ha denotes host-adapted clone.

c Other tissues include heart, bladder and tibiotarsal joint.

In order to assess whether mice exposed to *B. burgdorferi* clones expressing a static form of VlsE could mount an immune response capable of preventing reinfection, a similar experiment was conducted with C3H mice that had cleared sVlsE-induced infection. As shown in Table 4, WT spirochetes were able to successfully reinfect mice originally infected with and cleared of the sVlsE clone (sVlsE-WT). Conversely, these mice were not reinfected by ΔVlsE (group sVlsE-ΔVlsE; Table 4), suggesting that antibodies against non-VlsE surface antigens were generated during initial infection with the sVlsE clone. As expected, mice infected with sVlsE were resistant to reinfection with the host-adapted sVlsE clone (sVlsE-sVlsE; Table 4), presumably due to a preexisting immune response developed specifically against the static form of VlsE expressed in this mutant clone. Taken together, the above experiments demonstrate for the first time that variable VlsE is absolutely required for *B. burgdorferi* to reinfect both immunologically VlsE-naïve and VlsE-exposed mice.

### Variable VlsE is sufficient for escape from *Borrelia*-specific immune sera

**Figure 5:**
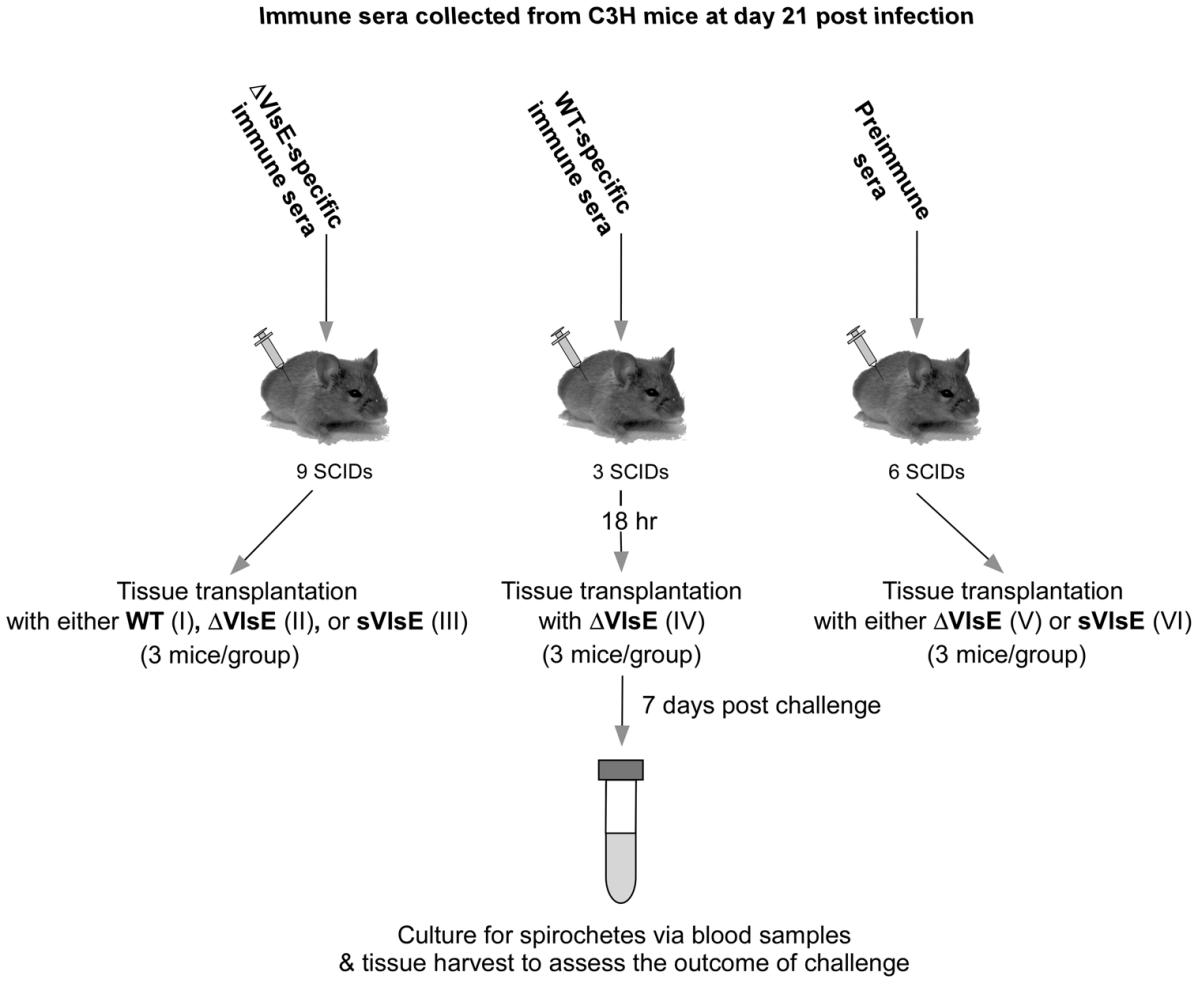
Experimental design to assay for a VlsE requirement for evasion of *B. burgdorferi*-specific antibodies. Immunologically-naïve SCID mice were injected with either WT-specific, ΔVlsE-specific, or preimmune sera. Mice were divided into groups of 3 animals each and challenged 18 hours later with host-adapted *B. burgdorferi* clones. Blood and other tissues were collected at day 7 post challenge and cultured for spirochetes. Group numbers are indicated in the parentheses.

Results from the above reinfection experiments suggest that variable VlsE is capable of providing protection against anti-*Borrelia* host immunity, specifically the adaptive immune response. To address whether variable VlsE prevented recognition of non-VlsE surface antigens by host *Borrelia*-specific antibodies, we designed a passive transfer experiment using SCID mice as a model. The experimental scheme included six groups of SCID mice as shown in Figure 5. Mice in groups I, II, and III received immune sera obtained from immunocompetent C3H mice that had cleared ΔVlsE-induced infection by day 21 post infection, and thus contained antibodies generated against various *B. burgdorferi* surface proteins except VlsE. Immunized SCID mice of groups I, II, and III were challenged with either host-adapted WT, ΔVlsE, or sVlsE spirochetes, respectively. At day 7 post transplantation, blood, heart, ear, bladder and joint tissues were harvested to determine whether the immune sera prevented infection. As expected, mice were successfully challenged by the WT clone despite the presence of ΔVlsE-specific immune sera (group I; Table 5). In contrast, the ΔVlsE clone was not detected in tissues of any mice, indicating that host-adapted spirochetes lacking the VlsE protein are susceptible to ΔVlsE-specific immune sera treatment (group II; Table 5). Consistent with the reinfection findings, ΔVlsE-specific sera also prevented host-adapted sVlsE infection in 3 out of 3 mice, suggesting that static VlsE does not allow *B. burgdorferi* to evade *Borrelia*-specific host antibodies (group III; Table 5).

**Table 5:** Infectivity of *B. burgdorferi* clones in passively-immunized SCID mice.

Animal group #	SCID mice injected with	SCID mice challenged with	Organs harvested at day 7 post challenge^a^					
			Total	Blood	Heart	Ear	Bladder	Joint
I	ΔVlsE-*specific sera*	WT	**3/3**	3/3	3/3	0/3	1/3	1/3
II	ΔVlsE-*specific sera*	ΔVlsE	**0/3**	0/3	0/3	0/3	0/3	0/3
III	ΔVlsE-*specific sera*	sVlsE	**0/3**	0/3	0/3	0/3	0/3	0/3
IV	WT-*specific sera*	ΔVlsE	**0/3**	0/3	0/3	0/3	0/3	0/3
V	*preimmune sera*	ΔVlsE	**3/3**	3/3	3/3	0/3	0/3	2/3
VI	*preimmune sera*	sVlsE	**3/3**	3/3	3/3	1/3	1/3	2/3

a Values listed correspond to numbers of cultures positive/numbers tested.

Immune sera generated from immunocompetent mice infected with the WT clone (WT-specific sera) also prevented challenge with the VlsE-deficient ΔVlsE clone (group IV; Table 5), suggesting that VlsE does not suppress production of non-VlsE specific antibodies in the course of an adaptive immune response. Expectedly, mice that received preimmune sera were successfully infected with either the ΔVlsE or sVlsE clone (groups V and VI; Table 5), demonstrating that these clones were fully capable of infecting mice. Together, these data support the findings of our reinfection experiments, emphasizing the importance of variable VlsE for *B. burgdorferi* evasion of the host adaptive immune response to *B. burgdorferi* surface antigens.

### Static VlsE-expressing *B. burgdorferi* can successfully resist immune sera treatment after established infection

One possible explanation for the inability of the sVlsE clone to reinfect VlsE-naïve mice is a low infectious dose of host-adapted spirochetes expressing the static form of VlsE. This could arise due to spontaneous loss of the lp28-1Δ*vls*::*vlsE* plasmid during the three-week infection period in SCID donor mice prior to tissue transplantation. Indeed, recent work in our lab has found that a portion of spirochetes recovered from tissues of SCID mice infected with the sVlsE clone no longer retain the lp28-1 plasmid (Rogovskyy and Bankhead, unpublished results). To account for the possibility of a low infectious dose of static VlsE-expressing clones, we allowed tissue-transplanted spirochetes to propagate *in vivo* for four days prior to immune sera treatment in order to increase the number of spirochetes carrying lp28-1Δ*vls*::*vlsE* during initial infection. Specifically, SCID mice were infected with either the host-adapted ΔVlsE or sVlsE clone (groups A and C; Table 6), and after 4 days post infection these mice were given immune sera obtained from immunocompetent mice that had cleared infection with ΔVlsE (ΔVlsE-specific sera). Infection was then monitored by culturing blood collected at day 3 and 7 post sera treatment.

**Table 6:** Treatment of infected SCID mice with ΔVlsE-specific immune sera.

Animal group	SCID mice infected with	Immune sera treatment with (day 4 post transplantation)	Blood taken post sera treatment at day^a^	
			3	7
A	ha ΔVlsE^b^	ΔVlsE-*specific sera*	**0/3**	**0/3**
B	ha ΔVlsE	*preimmune sera*	**3/3**	**3/3**
C	ha sVlsE	ΔVlsE-*specific sera*	**1/3**	**3/3**
D	ha sVlsE	*preimmune sera*	**3/3**	**3/3**

a Values listed correspond to numbers of cultures positive/numbers tested.

b ha denotes host-adapted clone.

The results show that ΔVlsE-specific immune sera treatment was able to successfully clear infection with host-adapted ΔVlsE spirochetes (group A; Table 6), verifying the bactericidal efficacy of the immune sera. In contrast, the sVlsE clone was able to survive in SCID mice despite the presence of ΔVlsE-specific immune sera, suggesting that these mutant spirochetes expressing static VlsE can evade host antibodies (group C; Table 6). The sVlsE isolates obtained from positive blood cultures were kanamycin resistant and PCR positive for *vlsE*, indicating that the lp28-1Δ*vls*::*vlsE* plasmid was retained in these recovered clones (data not shown). All mice infected with host-adapted ΔVlsE or sVlsE and given preimmune sera remained infected at day 3 and 7 post sera treatment, demonstrating the viability and infectivity of these clones under the experimental conditions (groups B and D; Table 6). Overall, these data suggest that static VlsE can provide some measure of immune avoidance for non-VlsE surface antigens, but only after infection is established.

### VlsE is dispensable for *B. burgdorferi* survival against passive immunization with T-cell independent antibodies

It has been previously shown that immune sera from *Borrelia*-infected T-cell deficient mice can protect naïve animals against a challenge with *B. burgdorferi* [40]. Although our data demonstrate a VlsE requirement for evasion of non-VlsE surface antigens from the host humoral immune response, it is unknown whether VlsE is also important to specifically evade T-cell independent (TI) antibodies. To address this, sera from *Borrelia*-infected Hsd∶Athymic Nude-*Foxn1^nu^* (nude) mice lacking functional CD4+ and CD8+ T cells was utilized as a source of *Borrelia*-specific TI antibodies.

To first determine whether variable VlsE is required to establish persistent infection in nude mice, naïve animals were infected with either host-adapted WT, ΔVlsE or sVlsE *B. burgdorferi* clones. Infection was monitored by sampling tissues at day 7, 14, 21, and 42 post infection. All nude mice were successfully infected with each of the different *B. burgdorferi* clones as determined by positive blood cultures taken at day 7 post infection (Table 7). Interestingly, all three clones were detectable from ear biopsies collected at day 14 and 21 post infection, indicating a greater ability of the ΔVlsE and sVlsE clones to persist in nude mice than immunocompetent C3H mice. In contrast to WT, however, ΔVlsE or sVlsE spirochetes could not be detected in ear tissues by day 42 post infection, suggesting that variable VlsE is required for long-term persistence in this tissue (Table 7). However, mutant spirochetes could be recovered from joint tissues, which suggests that this tissue site in nude mice may provide a protective niche for the VlsE-mutant clones.

**Table 7:** Infectivity of host-adapted VlsE mutants in naïve nude mice.

Tissue collected (at day post infection)	Naïve nude mice infected via tissue transplantation with^a^		
	ha WT^b^	ha ΔVlsE	ha sVlsE
Blood (day 7)	**3/3**	**3/3**	**3/3**
Ear (day 14)	3/3	3/3	2/3
Ear (day 21)	3/3	2/3	1/3
Ear (day 42)	3/3	0/3	0/3
Joint (day 42)	**3/3**	**3/3**	**3/3**

a Values listed correspond to numbers of cultures positive/numbers tested.

b ha denotes host-adapted clone.

**Figure 6:**
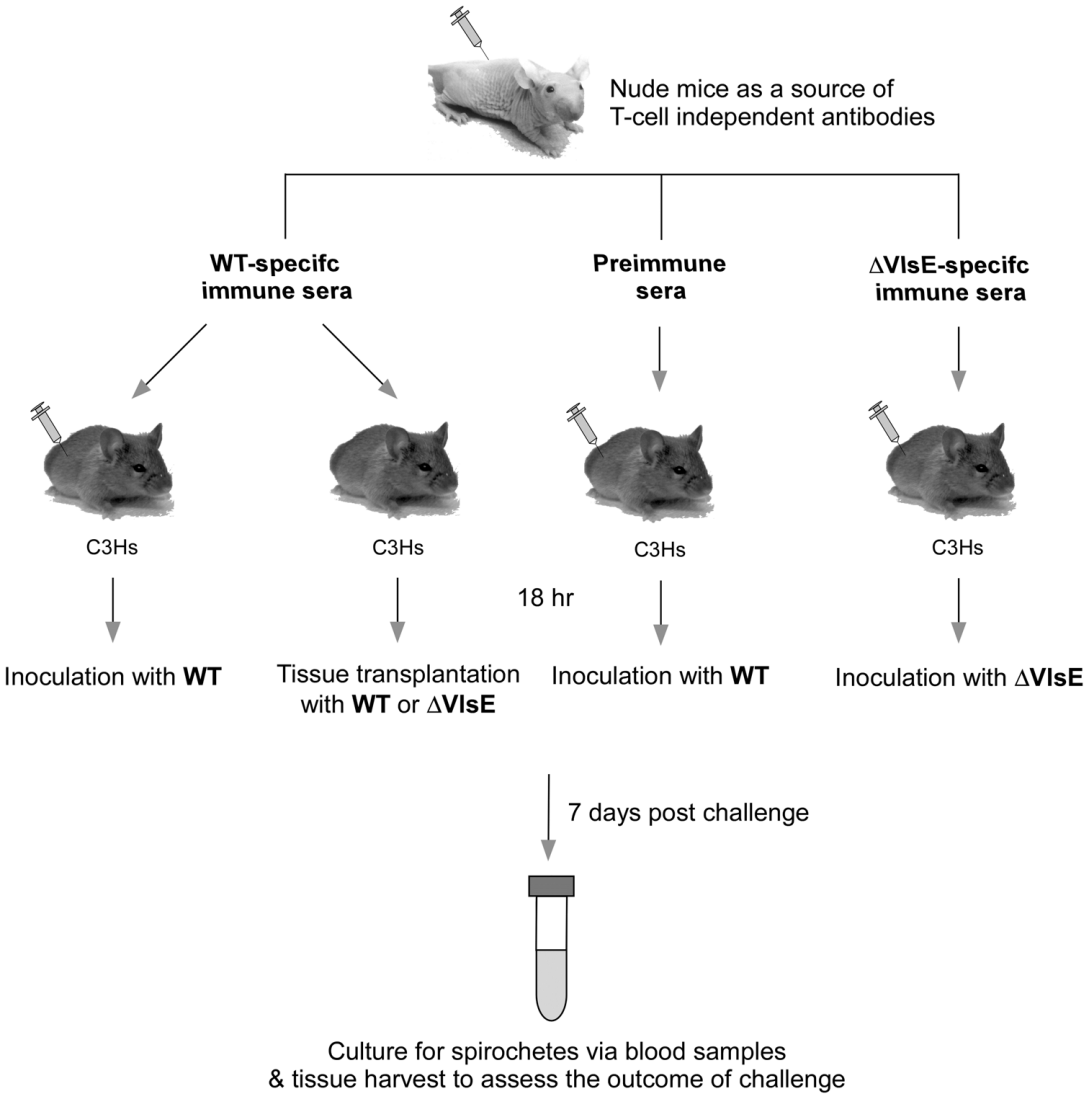
Experimental design to assay for a VlsE requirement for evasion of T-cell independent antibodies. Immunologically-naïve C3H mice were injected with either WT-specific, ΔVlsE-specific, or preimmune sera originated from nude mice. The sera-treated animals were divided into groups of 3 each and challenged 18 hours post-sera treatment with either host-adapted or *in vitro*-grown *B. burgdorferi* clones. Blood and other tissues were harvested at day 7 post challenge and cultured for spirochetes. Group numbers are indicated in the parentheses.

To address whether VlsE is important to specifically evade TI antibodies, immunologically-naïve C3H mice were passively immunized with immune sera containing *Borrelia*-specific TI antibodies and then challenged with either the WT or ΔVlsE clone (Figure 6). To obtain TI immune sera, nude mice infected with either WT or ΔVlsE spirochetes were bled at day 14 post infection and sera collected. This time point was chosen to ensure that all nude mice were no older than 8 weeks of age at the time of sera collection; older nude mice have been shown to be somewhat “leaky” and exhibit low-level T cell production [41]. The infection was monitored by culturing blood sampled at day 7 post infection.

Consistent with a previous study [40], WT-specific sera was able to prevent infection by *in vitro*-derived WT spirochetes (Table 8, group 1). In contrast, 3 out of 3 animals that received preimmune sera became infected with this *in vitro*-derived WT clone, verifying that these spirochetes were fully infectious at the time of challenge (Table 8, group 2). The total IgM concentration present in the immune sera used above was found to be approximately 38 ng as determined by ELISA, and was utilized in all subsequent experiments described below.

**Table 8:** Infectivity of *B. burgdorferi* clones in C3H mice passively immunized with T-cell independent antibodies.

Animal group #	C3H mice injected with immune sera taken from nude mice	C3H mice challenged with	Total^a^
1	WT-*specific sera*	WT	0/4
2	*preimmune sera*	WT	3/3
3	WT-*specific sera*	ha WT^b^	3/3
4	WT-*specific sera*	ha ΔVlsE	4/4
5	ΔVlsE-*specific sera*	ΔVlsE	4/4

a Values listed correspond to numbers of cultures positive/numbers tested.

b ha denotes host-adapted clone.

C3H mice that received WT-specific TI sera were also challenged with host-adapted WT (Table 8, group 3), and results show that the immune sera was unable to prevent infection by these spirochetes. Taking into account that *vls* recombination does not detectably occur during *in vitro* cultivation, but SCID-derived host adapted WT will have experienced some level of *vlsE* antigenic variation [18], this finding may suggest that variable VlsE is necessary to evade the TI antibody response. Alternatively, low expression of VlsE and/or the presence of other surface antigens normally downregulated in host-adapted spirochetes may explain the inability of *in vitro*-derived WT *B. burgdorferi* to successfully challenge TI sera-treated mice. However, both host-adapted and *in vitro*-derived ΔVlsE spirochetes were also able to establish infection in mice (Table 8, group 4 and 5) despite the presence of TI immune sera. Together, these findings suggest that VlsE is not necessary for *B. burgdorferi* to survive WT-specific TI antibodies, and that the immune sera contained non-borreliacidal levels of TI antibodies to surface antigens other than VlsE. Moreover, the finding that *in vitro*-derived WT spirochetes are susceptible to TI antibodies, while *in vitro*-derived ΔVlsE clones are not, is suggestive that the TI antibody response may be directed mainly to VlsE.

## Discussion

### Requirement of VlsE for host reinfection

Reinfection by *B. burgdorferi* has been recognized in both humans and canines [5], [13], [42], [43]. Previous studies using a murine model have demonstrated that cured mice displayed short-term immunity to reinfection by tick transmission, intradermal *B. burgdorferi* inoculation, or with autologous infected tissues [44], [45]. It was presumed that because these *B. burgdorferi* isolates were autologous, their inability to reinfect was most likely due to a developed immunity to antigenically similar spirochetes. Yet, a contemporary study demonstrated that mice passively immunized to *B. burgdorferi* were protected against *in vitro*-derived spirochetes but susceptible to challenge by host-adapted spirochetes [37]. Another study showed that mice that had cleared the VlsE-deficient ΔVlsE clone, and thus were immune to spirochetes antigenically distinct from wild-type *B. burgdorferi*, were also resistant to reinfection by *in vitro*-derived wild-type spirochetes [24]. Because the level of VlsE expression is substantially greater during murine infection than that of *in vitro*-grown spirochetes [35], [46], the possibility was raised that the failure to establish host reinfection could potentially be accounted for by a lack of VlsE upregulation [24].

In the present study, host-adapted clones of *B. burgdorferi* were used in order to eliminate potentially reduced VlsE expression levels that have plagued previous studies. The data presented here demonstrate that host-adapted WT expressing variable VlsE is able to reinfect immunocompetent VlsE-naïve and VlsE-exposed C3H mice. Conversely, a *B. burgdorferi* VlsE-deficient mutant clone was shown to be unable to reinfect these same mice. The sVlsE clone capable of expressing only a static form of VlsE was also found to be unable to reinfect mice, suggesting that the variable form of VlsE is necessary to allow host reinfection. Finally, passive transfer experiments demonstrated that immune evasion of non-VlsE surface antigens was mediated against host *Borrelia*-specific antibodies. Together, the above experiments demonstrate the absolute requirement of variable VlsE for reinfection by the Lyme disease pathogen.

A possible explanation for the inability of the sVlsE clone to reinfect mice is potentially low numbers of VlsE-expressing spirochetes in the transplanted SCID ear tissues. In support of this, the sVlsE clone expressing a static form of VlsE could successfully challenge sera-treated mice, but only after infection was first established (day 4 post infection) presumably because this allowed expansion of VlsE-expressing spirochetes prior to sera treatment. However, an additional factor that may have contributed to the failure of the sVlsE clone to reinfect is delayed and/or low-level expression of VlsE in the murine host. Western blot analysis showed that *in vitro*-cultured sVlsE mutant spirochetes expressed VlsE at lower levels than those of the wild type (see Fig. 2). Transcriptional regulation is thought to involve the 5′-noncoding region of *vlsE* containing an inverted DNA repeat that is predicted to form a stable cruciform structure [47]. Though the inverted repeat does not seem to limit access to the *vlsE* promoter *in vitro* [48], it still may be potentially involved in timely transcriptional upregulation of *vlsE* in the mammalian host. The *vlsE* gene copy in our generated sVlsE mutant does not contain this region due to the inherent difficulties in cloning large DNA inverted repeats. Therefore, it is possible that expression of VlsE in the SCID-derived host adapted sVlsE clone remained at insufficient levels for spirochetes to evade an antibody-mediated immune response in order to reinfect C3H mice. Future studies using sVlsE clones containing the full-length inverted DNA repeat sequence will be necessary to address this question.

An alternative explanation for the inability of the ΔVlsE clone to reinfect mice and resist *Borrelia*-specific immune sera is the presence of antibodies developed to outer surface protein C (OspC). OspC is an immunodominant lipoprotein whose expression is required for tick-derived or *in vitro*-grown *B. burgdorferi* to establish infection *in vivo* [49], [50], [51], [52], [53], [54]. It has been hypothesized that VlsE may assume an essential, yet unknown function of OspC [55]. If true, then the expectation would be that VlsE-deficient spirochetes will only survive in mammals if OspC continues to be expressed. Thus, it is conceivable that OspC-specific immunoglobulins could readily eliminate OspC-expressing spirochetes, whereas non-OspC expressing clones simply fail to establish infection due to the absence of OspC and its putative substitute, VlsE. However, this possibility fails to completely explain the inability of the sVlsE clone to reinfect VlsE-naïve mice. Experiments addressing the role of anti-OspC antibodies in this process are currently underway.

### VlsE-mediated immune avoidance in *B. burgdorferi*?

The data presented here suggest that variable VlsE is necessary for *B. burgdorferi* to specifically evade the acquired humoral immune response. Passively-transferred antibodies that were developed to non-VlsE surface antigens and shown to provide immunity against the VlsE-deficient clone, were not borreliacidal to wild-type spirochetes. This VlsE-mediated immune evasion may hint at a possible shielding mechanism that has been previously proposed for preventing other surface antigens of *B. burgdorferi* from being recognized by borreliacidal antibodies [24], [32], [33], [34]. However, the experiments detailed here do not offer much insight into the mechanistic details of how VlsE may shield surface epitopes. A precedent for potential antibody shielding by *B. burgdorferi* has been previously demonstrated for the outer surface protein, OspA. This lipoprotein, synthesized in ticks and rarely in mammals [56], [57], was shown to limit the access of antibodies to the immunogenic protein P66 *in vitro* [34]. Additionally, a more recent study demonstrated that OspA expressed in feeding ticks blocked recognition of conserved borrelial antigens by host antibodies derived during the uptake of a blood meal [58].

It is also plausible that VlsE could mask surface antigens in association with other molecules. It has been shown that *Plasmodium falciparum* can evade an immune response through IgM shielding of protective IgG epitopes [59]. Interestingly, the binding of non-specific IgM shielded surface proteins without compromising their function [59]. A similar immunoevasive strategy could conceivably be in place for VlsE-mediated shielding, whereby non-borreliacidal immunoglobulins or other host factor(s) may bind specifically or non-specifically to the highly antigenically variable VlsE protein.

Another possible mechanism of VlsE-mediated immune evasion is linked to the immunodominant nature of this lipoprotein [35]. VlsE may override antibody production that leads to predominant levels of anti-VlsE antibodies and subdominant titers of immunoglobulins to other surface antigens [21]. However, data presented here suggest that VlsE does not suppress production of non-VlsE specific antibodies in the course of an adaptive immune response. The immune sera generated in immunocompetent mice against wild-type *B. burgdorferi* contained non-VlsE specific immunoglobulins at levels sufficient enough to prevent infection by the ΔVlsE clone (see group IV; Table 5). Yet, the findings themselves do not completely exclude an “overriding” mechanism, but instead only argue against such a system for evading an acquired humoral response.

Previous studies have suggested that VlsE may act as a TI antigen [24], [33], possibly through direct stimulation of IgM-producing B cell subsets. Therefore, it remains possible that VlsE may function to override TI antibody production during the very early stages of infection. Our data suggest that immune sera derived from nude mice infected with wild-type *B. burgdorferi* contained subdominant titers of TI antibodies against non-VlsE surface antigens, as shown by their inability to prevent infection by the ΔVlsE clone. In contrast, the same sera appeared to have anti-VlsE TI antibodies at sufficient levels to prevent infection by *in vitro*-grown wild-type *B. burgdorferi* (Table 7).

In conclusion, the work presented here demonstrates for the first time the absolute requirement of variable VlsE for host reinfection. Moreover, the data suggest that VlsE is specifically involved in evasion of non-VlsE surface antigens from the acquired humoral immune response. Future study into the mechanism behind VlsE-mediated immune avoidance has the potential to reveal how *B. burgdorferi* resists the robust antibody response elicited against surface antigens other than VlsE. Obtaining such knowledge could significantly improve our understanding of immune evasion by this important pathogen, and may have general implications for other pathogen systems as well.

## Materials and Methods

### Ethics statement

All experimental procedures involving animals were carried out in accordance with the American Association for Accreditation of Laboratory Animal Care (AAALAC) protocol and the institutional guidelines set by the Office of Campus Veterinarian at Washington State University (Animal Welfare Assurance A3485-01 and USDA registration number 91-R-002). Washington State University AAALAC and institutional guidelines are in compliance with the U.S. Public Health Service Policy on Humane Care and Use of Laboratory Animals. All animals were maintained at Washington State University in an AAALAC-accredited animal facility. The Washington State University Institutional Animal Care and Use Committee reviewed and approved the animal protocol associated with the present study.

### Murine infection

Male, C3H/HeNHsd (C3H) and Hsd∶Athymic Nude-*Foxn1^nu^* (nude) mice of 4–6 weeks of age were obtained from Harlan (Indianapolis, IN), and C3SnSmn.CB17-*Prkdc^scid^*/J (SCID) mice of 4–5 weeks of age were purchased from Jackson Laboratories (Bar Harbor, ME). C3H, SCID, and nude mice of 5–8 weeks old were challenged through needle inoculation with 1×10^3^ cells ml^−1^ and 1×10^4^ cells ml^−1^ via intraperitoneal and subcutaneous routes, respectively. All *B. burgdorferi* clones harboring recombinant plasmids were cultured in media containing appropriate antibiotic prior to murine infection. *B. burgdorferi* clones were passaged no more than two times *in vitro* from frozen glycerol stock prior to use in mouse infection studies.

Alternatively, animals were challenged by tissue transplantation of ears derived from infected SCID mice as previously described [44]. For tissue transplantation, SCID mice were sacrificed at day 28 post challenge and ear pinnae were cut into small, circular pieces by a sterile ear punch and stored at −80°C. Infected ear tissues were thawed on ice as needed and three ear pieces per animal were used for a mouse challenge. Specifically, a skin incision (2–3 mm) was aseptically made in the lumbar region and ear pieces were inserted subcutaneously. The infectivity of tissue-derived spirochetes was tested on naïve C3H mice (Table 3). To verify infection, 50 ul of blood was aseptically drawn from a mouse via maxillary bleed at day 4 or 7 post challenge and cultured in 3 ml of BSK-II containing *Borrelia* antibiotic cocktail (0.02 mg ml^−1^ phosphomycin, 0.05 mg ml^−1^ rifampicin and 2.5 mg ml^−1^ amphotericin B). To monitor the progress of infection, ear, heart, bladder, and joint tissues were aseptically harvested at various time points post challenge and cultured in 1.0 ml of BSK-II supplemented with the antibiotic cocktail. Dark-field microscopy was used to confirm the presence of viable spirochetes for each cultured tissue. For each use, infected ear tissue was tested for the presence of viable borrelial cells by culture.

### Bacterial strains

*Borrelia burgdorferi* strain B31-A3 (WT) was kindly provided by Patti Rosa. B31-A3Δ*vls* (ΔVlsE) was generated in a previous study [24], and was a generous gift from George Chaconas. All *B. burgdorferi* clones were cultivated in liquid Barbour–Stoenner–Kelly II media (BSK-II) supplemented with 6% rabbit serum (Cedarlane Laboratories, Burlington, NC) and incubated at 35°C.

### Plasmid construction

To obtain the *B. burgdorferi* lp28-1Δ*vls*::*vlsE* clone (sVlsE), a targeted deletion plasmid, pAR2, was generated. The target region identical to DNA basepair coordinates 17,296 to 18,800 on lp28-1 was PCR amplified using the P266 (CCGGGGTACCGCTGTATAATGTC AAATGGCTAGG) and P267 (GTGCCGCTCGAGAGGCTGCTGATGAGGCGAG) primers. The amplicon was cloned into pJET1.2 vector using CloneJET PCR Cloning Kit (Fermentas, USA). The target sequence was then recovered by digestion with KpnI and XhoI, followed by cloning into pTB44 [24] to generate pAR1. pTB44 contains a *flgBp*-driven *kan* gene and a 70 bp replicated telomere from the left end of lp17. The *vlsE* expression site with native promoter was PCR amplified from pMBL20 (a gift from Steven Norris; [23]) using the P260 (GGTCTAGAAGAAATGAAAAATTCTCTCACCTACACTT) and P261 (GCCGGCCGGAGGG CATAGTCGTGTCCATACA) primers, and then cloned into pJET1.2. The *vlsE* insert was recovered by digestion with EagI and XbaI and cloned into pAR1.

### *B. burgdorferi* transformation

*B. burgdorferi* cells were electroporated with pAR2 as previously described [24]. After electroporation with a total of 50 ug of DNA, spirochetes were resuspended in 10 ml prewarmed BSK-II media. Cells were recovered at 35°C for 24 hr and then diluted in 100 ml of pre-warmed BSK-II supplemented with 200 ug ml^−1^ kanamycin. The transformed cell suspension was aliquoted into 96-well plates and incubated at 35°C for 3–4 weeks. DNA from positive wells was extracted using a DNeasy Blood and Tissue Kit (Qiagen, Germantown, MD), and used for PCR analysis to confirm the presence of the kanamycin-resistance gene and *vlsE* utilizing P54/55 (CATATGAGCCATATTCAACGGGAAACG and AAAGCCGTTTCTGTAA TGAAGGAG) and P243/244 (GCGATATAAGTAGTACGACGGGGAAACCAG and CAAGGC AGGAGGTGTTTCTTTACTAGCAGC) primer sets, respectively. Plasmid content for each verified transformant was determined by PCR using plasmid-specific primers as previously described [39].

### Field inversion gel electrophoresis

Plasmid DNA from *B. burgdorferi* transformations was purified utilizing the Qiagen Plasmid Midi Kit (Qiagen, Germantown, MD). Approximately 450 ng of DNA was separated on a 0.65% Seakem agarose gel at 80 V for 40 min, followed by initiation of Program 0 on an MJ Research PPI-200 programmable power inverter (kindly provided by D. Scott Samuels) at 80 V for 21 hr with buffer recirculation [60].

### Immunoblotting

*B. burgdorferi* clones were grown in BSK-II to the late stationary phase. Cells were counted, pelleted by centrifugation at 6,000×g for 10 min at 4°C, and then washed twice with ice-cold PBS. After removal of PBS, the cells were suspended in sodium dodecyl sulfate (SDS)-polyacrylamide gel electrophoresis sample buffer (100 mM Tris [pH 6.8], 2% SDS, 5% β-mercaptoethanol, 10% glycerol, 0.01% bromophenol blue), and incubated at 95°C for 10 min. Approximately 1×10^6^ or 1×10^8^ cells were loaded to each sample lane of 15% acrylamide minigel (see SDS-PAGE analysis in Figure S1). Resolved proteins were transferred onto polyvinylidene fluoride (PVDF) membrane with a pore size of 0.45 um (Immobilon-P, Millipore, USA). The blot was blocked with 5% nonfat dry milk in PBS for 18 hr at 4°C and then incubated in the same solution supplemented with either 1∶1,000 diluted rabbit anti-FlaB antibody (Rockland Immunochemicals, Gilbertsville, PA), 1∶2,000 diluted rabbit anti-C_6_ serum (generously provided by Mario Philipp), or 1∶1,000 diluted mouse anti-WT, -sVlsE, or -ΔVlsE immune, or preimmune sera for 1 hr. To generate anti-WT, -sVlsE, or -ΔVlsE immune sera, naïve C3H mice (3 mice per group) were infected with WT, sVlsE, or ΔVlsE (1.1×10^4^ cells ml^−1^) via needle inoculation. At day 28 post infection blood was collected and equal amount of immune sera derived from animals of the same group were pooled and filter-sterilized by passage through 0.22 um syringe filter. Preimmune sera were collected from three naïve C3H mice.

After 4 washes of 10 min each with TBST, the primary antibodies were detected utilizing donkey anti-rabbit or anti-mouse HorseRadish Peroxidase (HRP)-conjugated secondary antibody (Jackson ImmunoResearch Laboratories, West Grove, PA) diluted to 1∶5,000 and 1∶1,000, respectively, in TBST for 30 min. The blot was washed 3 times in TBST for 10 min each, followed by a last wash in nano-pure water. The blots were visualized on Enhanced ChemiLuminescence (ECL) development. Surface proteolysis involving proteinase K digestion of intact spirochetes was performed as previously described [61].

### Passive immunization of mice

To obtain immune sera, C3H and nude mice were infected with *B. burgdorferi* through needle inoculation or tissue transplantation as described above. Blood from isoflurane-euthanized, infected C3H and nude mice were collected via cardiac puncture at day 21 and 14 post infection, respectively. Approximately, 600–900 ul of immune serum was collected from each mouse. Collected blood was kept at room temperature for 30 min and then centrifuged at 6,000×g for 15 min. Sera were removed from the blood cell pellet and stored at −20°C until required for passive immunization. Immune sera were thawed on ice immediately before use and pooled if obtained from mice infected with the same clone. Sera were then diluted to 1∶3 with sterile saline to minimize a loss associated with sterilization and immunization. Diluted sera were filter-sterilized by passage through 0.22 um syringe filter. The mice to be passively immunized were injected with total of 300 ul of diluted immune sera. IgM titer was measured by a mouse IgM ELISA according to the manufacturer's instruction (Immunology Consultant Laboratory, OR). *B. burgdorferi*-specific sera derived from nude mice at day 14 post infection contained 377 ng/ml of IgM.

## Supporting Information

Figure S1 **SDS-PAGE analysis of whole-cell lysates of** ***B. burgdorferi*** **clones.** WT, sVlsE, and ΔVlsE whole-cell lysates (10^6^ cells/lane) were subjected to electrophoresis in a 15% sodium dodecyl sulfate polyacrylamide gel under reducing conditions. Electrophoresis was carried out in Tris-glycine buffer containing 0.01% SDS. The slab gel was stained with Coomassie Blue R-250 and destained with methanol∶water∶acetic acid (5∶4∶1, v/v).
(TIF)
