# Supplementary figures and images for: Variable VlsE Is Critical for Host Reinfection by the Lyme Disease Spirochete
Source: PLoS One. 2013 Apr 8;8(4):e61226. doi: 10.1371/journal.pone.0061226 (PMC3620393; doi:10.1371/journal.pone.0061226)

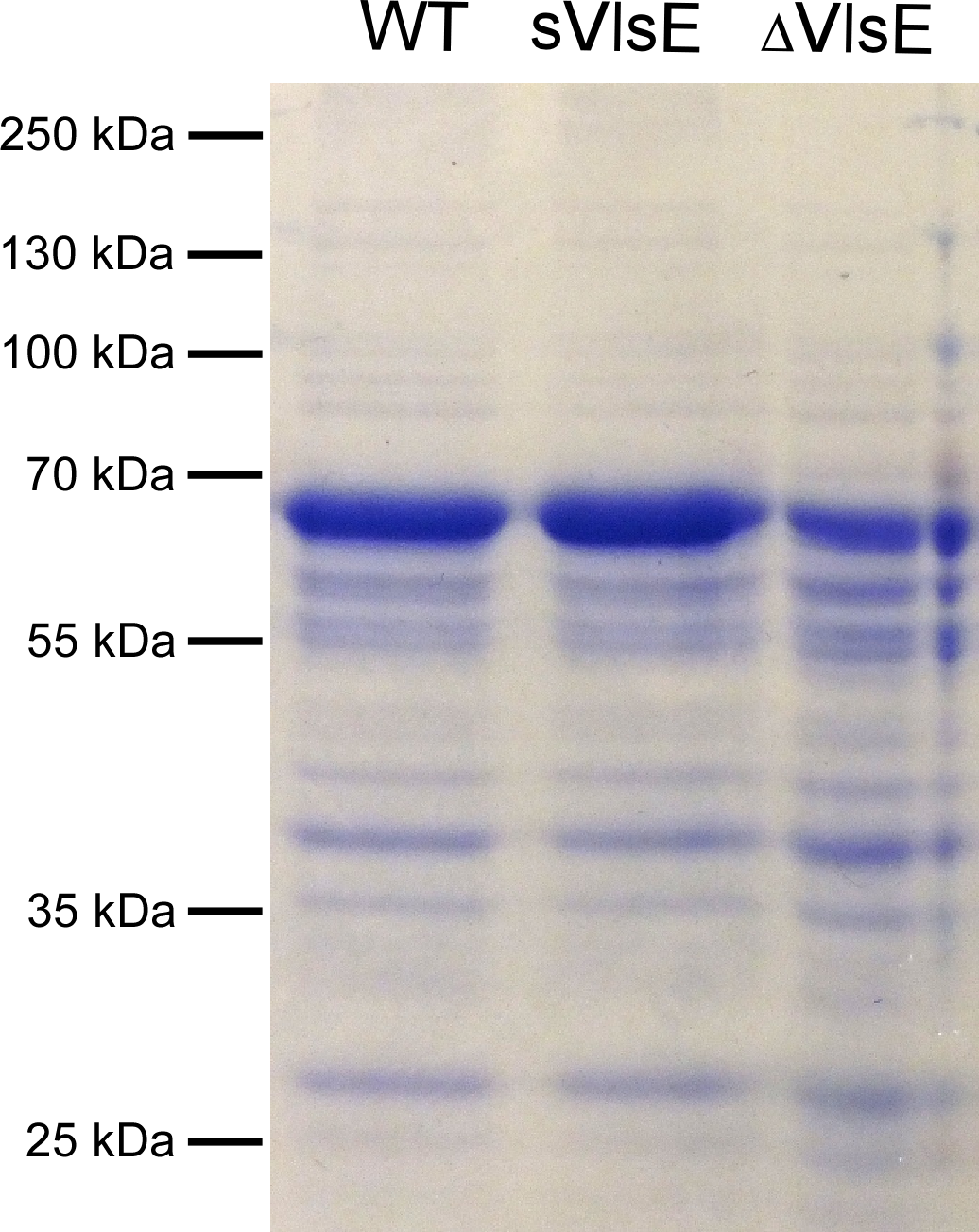

Supplement: Figure S1 — SDS-PAGE analysis of whole-cell lysates of B. burgdorferi clones. WT, sVlsE, and ΔVlsE whole-cell lysates (106 cells/lane) were subjected to electrophoresis in a 15% sodium dodecyl sulfate polyacrylamide gel under reducing conditions. Electrophoresis was carried out in Tris-glycine buffer containing 0.01% SDS. The slab gel was stained with Coomassie Blue R-250 and destained with methanol∶water∶acetic acid (5∶4∶1, v/v). (TIF) [file pone.0061226.s001.tif]
